# 

**Published:** 1896-08-22

**Authors:** 


					x\ u
r THE HOSPITAL. ? {ABSOLUTELY PURE, therefore BEST, August 22kd, 1896.
CADBURY'S U fe* CADBURY'S
COCOA . >HP' IJoil COCOA
"The standard of highest purity at present ^   ' NO ALKALIES USED.
attainable."?Lancet.
LASQonr Western ItLEiaMART
No. 517. 4Vol. XX.
oattjrday,
August 22nd, 1896.
iAJiofiOR tY/CH HOSPl.TAJ. %
WITH EXTRA NURSING SUPPLEMENT. pb-ice twopence.
[Registered at the General Post Office as a Newspaper for MontMvParta 6d ? tinttfr^n 1
transmiflfcion in the United Kingdom and Abroad,] Mto per&VsV. ftMJ.

				

